# Synthesis of polydentate, multi metal ion sensing, unsymmetrical Schiff bases with complimented antifungal activity

**DOI:** 10.55730/1300-0527.3412

**Published:** 2022-03-01

**Authors:** Saranya DHASARATHAN, Shunmugaperumal SELVARAJ, P. Kamatchi SELVARAJ

**Affiliations:** PG & Research Department of Chemistry, Government Arts College for Men (Autonomous), Nandanam, Tamil Nadu, India

**Keywords:** Azomethine, cation sensors, binding attitude, molecular docking, MLCT band

## Abstract

Polydentate, unsymmetrical, and multi metal ion sensing Schiff bases comprised of ferrocenecarboxaldehyde attached azomethine group at one side and aromatic aldehyde linked imine on the other side have been synthesized. Cumulative addition of different metal salts solution to receptors solution, changes the electronic spectra contrarily and for the addition of Cu^2+^ ions, generation of MLCT charge transfer band responsible for the coordination of metal ion with a receptor is observed. Electrochemical data (ΔE_p_) arrived from the cyclic voltammograms suggest a quasi-reversible process. The modest concentration of metal ions required for effective sensing by the sensory material is calculated from the I_pa_ values observed for metal ion added and metal free sensor solutions. Inhibition zones noticed in in vitro analysis for two fungi, two gram positive and two gram negative bacterial stains interpret that the new compounds possess high antifungal activity. Binding energy calculation using molecular docking software also ascertains the antifungal bustle.

## 1. Introduction

Development of sensing materials producing different color or giving fluorescence at dissimilar wavelengths upon the combination, either with various essential metal ions involved in a biological activity or nonessential metals that are hazardous to health is a rising field of interest [1] to the scientist working in the area of medicine, environment, material science, chemical and biological sciences [2]. The Volume of work has been published about the synthesis of cations & anions sensing Schiff base ligands [3–5] due to their simplicity in synthesis, ability to recognize analytes, selectivity along reproducibility, and minimum time & technical requirements [6]. The tetra or pentadentate nature of Schiff bases made them chelating ligands in coordination chemistry [7, 8].

Accumulation of a trivial amount of nonessential Hg^2+^ ions in the biological system damages the neurological organization, kidney and leads to malfunctions of the brain [9]. The role of Cu^2+^ ions in the mechanism of oxygen transfer and cellular energy manufacture projected copper as an essential metal [10]. Prescribing copper-containing drug for copper deficiency is under practice [11]. Excess intake of copper changes the metabolic activities of enzymes and causes depression, Alzheimer’s disease, vomiting, infertility, and miscarriages [12]. Realization of respiratory problems, lung cancer, and glitch in the nervous system has been reported for overconsumption of Ni^2+^ ions [13]. In addition to cancer stimulation, Cd^2+^ ingestion causes changes in calcium metabolism and pulmonary edema [14]. Softness at the bone joint, delay in the attainment of sexual maturity in adolescents, and diminishment of brain progression in children is reported for the consumption of Pb^2+^ ions [15]. Reduction in the level of essential metal Mn^2+^ affects the level of sugar in blood, immune and nervous systems [16]. Surplus accumulation of Mn^2+^ is linked with Parkinson’s disease [17].

Synthesis of unsymmetrical, Schiff bases N’^1^-((E)-ferrocenylidene)-N’^3^-((E)-2-nitrobenzylidene)malonohydrazide and N’^1^-((E)-ferrocenylidene)-N’^3^-((E)-2-hydroxy-5-nitrobenzylidene))malonohydrazide comprising of ferrocene group at one side and normal aromatic segment on the other side of leading malonyldihydrazide structure is discussed in this paper. Sensing aptitude of the newly synthesized compound towards Hg^2+^, Mn^2+^, Pb^2+^, Cd^2+^, Ni^2+^, and Cu^2+^ is also itemized through this work.

## 2. Experimental

### 2.1. Materials

Analytical grade chloride salts of Mn, Hg, Ni & Cu, and acetate salts of Cd & Pb purchased from Sigma–Aldrich were used in spectral and electrochemical studies. For synthesis and purification of receptor, analytical grade ferrocenecarboxaldehyde, 2-nitrobenzaldehyde, 2-hydroxy-5-nitrobenzaldehyde, dimethylmalonate, hydrazinehydrate, and silica gel were procured from E. Merck industry. HPLC grade acetonitrile acquired from E-Merck and spectral grade ethanol got from Commercial Alcohols, Canada was employed in sensing studies. Supporting electrolyte Tetrabutylammoniumperchlorate (99+% purity) received from Chemical Center, Mumbai was utilized (under care) as such in CV studies.

### 2.2. Instruments

Perkin-Elmer 337 spectrometer was used to record FTIR spectrum with KBr pellets in the range of 400–4000 cm^−1^. Bruker Daltonics esquire 3000 spectrometer was engaged to observe the mass spectra. BRUKER AVANCE spectrometer (500 MHz) was employed to detect the ^1^H NMR spectra using C_2_D_5_OD as solvent. Bruker Avance 400 MHz NMR spectrometer was utilized to record the ^13^C NMR spectrum in DMSO solvent. SHIMADZU MODEL UV-1800 240V spectrophotometer was utilized to perceive the UV–visible spectra between 200 and 800 nm. CHI electrochemical analyzer 1200B model having Ag/AgCl as reference electrode, glassy carbon as working electrode and platinum as the counter electrode was exploited to identify the cyclic voltammograms. CV studies were carried out in nitrogen atmosphere using 0.1 M tetrabutylammonium perchlorate as a supporting electrolyte. Perking – Elmer 2400 series CHSN/O analyzer was hired to estimate the C, H, and N contents.

### 2.3. Synthesis of N’^1^-((E)-ferrocenylidene)-N’^3^-((E)-2-nitrobenzylidene)malonohydrazide [R1]

The precursor compound malonyldihydrazide was synthesized [18] by allowing 1 mole of diethyl malonate to react with 2 moles of hydrazine hydrate. Recrystallized malonyldihydrazide was used to prepare 50 mL of 0.01 molar ethanol solutions. Another solution containing 0.01 mole of 2-nitrobenzaldehyde and 0.01 mole of ferrocenecarboxaldehyde in 150 mL ethanol was added to the above solution with stirring for half an hour and then refluxed for 6–7 h (Scheme). After cooling, the reaction mixture was concentrated to get reddish-yellow colored N’^1^-((E)-ferrocenylidene)-N’^3^-((E)-2-nitrobenzylidene)malonohydrazide. Purification was carried out in a silica gel column using ethanol as eluent. Color: Dark reddish-orange. Yield: 0.4172 g (81%), m.p. 80 °C.

### 2.4. Synthesis of N’^1^-((E)-ferrocenylidene)-N’^3^-((E)-2-hydroxy-5-nitrobenzylidene))malono hydrazide [R2]

In the place of 2-nitrobenzaldehyde, 2-hydroxy-5-nitrobenzaldehyde was employed to prepare the compound N’^1^-((E)-ferrocenylidene)-N’^3^-((E)-2-hydroxy-5-nitrobenzylidene)) malonohydrazide [R2] and all other steps followed in the synthesis of compound R1 was adopted sequentially (Scheme). Colour: Dark reddish-orange. Yield: 0.4812 g (74%), m.p. 85 °C.

### 2.5. In vitro antimicrobial activity

Standard procedure reported in the literature [19] for in vitro antimicrobial studies was exploited to expose the antifungal activity against fungi and antibacterial activity against two gram positive & two gram negative bacteria. An average value obtained from three different antimicrobial processes was considered for analysis.

### 2.6. Molecular docking studies

To investigate the binding mode of R1 and R2 with the target proteins, Autodock 4.2.6 version [20] running on Windows 7 was used. Protein extracted from Research Collaboratory for Structural Bioinformatics (www.RCSB.org) data Bank (PDB) provided the enzymes used for docking studies. MGL tools (Molecular Graphics Laboratory) of Autodock were used to get the docking score. The structures of compounds R1 and R2 were drawn using ChemSketch and converted to 3D structure with the help of 3D optimization tool. Geometrical optimization of the ligands structures was carried out by ligand module of Molecular Mechanics Force Field 94 (MMFF94). Thereafter, the docking position of prepared ligands with preferred proteins of the enzymes was computed by engaging Autodock tools and best docked pose was analyzed.

## 3. Results and discussion

### 3.1. Elemental and mass spectral analysis

Elemental analysis data of the synthesized compounds matches very well with the theoretical value: Anal.Calc (%) for R1,(C_21_H_19_N_5_O_4_Fe): C,54.78; H,4.13; N, 15.21; O,13.91 Found: C,54.74; H,4.8; N, 15.12; O,13.67. For R2, (C_21_H_19_N_5_O_5_Fe): C,52.9; H,3.99; N,14.7; O,16.8 Found: C,52.2; H,3.91;N,14.2;O,16.3. Appearance of molecular peak (ESI) m/z at 460 for R1 and 476 for R2 (Figure S1 and S2) on mass spectral analysis also ascertain the formation of expected receptors.

### 3.2. FTIR spectral analysis

**Figure 1:**
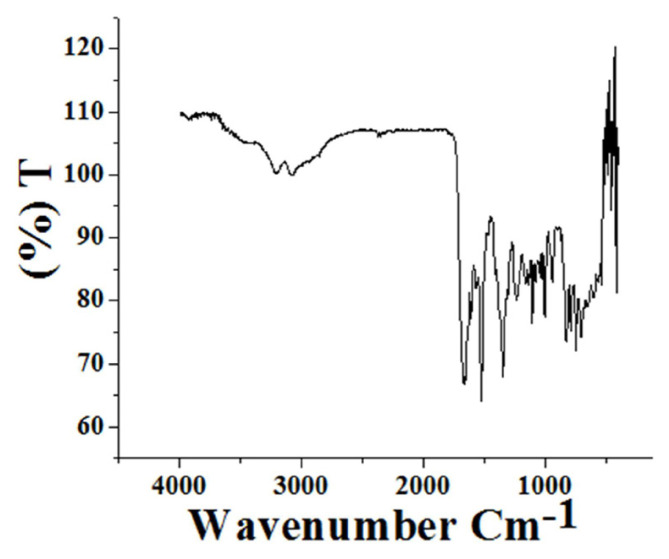
Spectrum showing the peaks appeared for R1 in FTIR analysis.

Ferrocene cyclopentadienyl ring tilt stretching vibration and C-H out of plane bend vibration appear at 472 cm^−1^ and 822 cm^−1^ respectively in the FTIR spectrum of R1 (Figure 1). The −C-C-H bending vibration in the cyclopentadienyl ring emerges between 939 cm^−1^ to 1227 cm^−1^ [21]. Stretching of −NO_2_, −CH=N (imine) and amide −C=O arises at 1344 cm^−1^, 1515 cm^−1^, and 1668 cm^−1^ respectively [22,23]. Peaks responsible for water of hydration and stretching vibration of secondary amine ascends near 3225 cm^−1^ and 3090 cm^−1^. FTIR spectrum of R2 (Figure S3) also contains all the above mentioned peaks and the phenolic-OH group stretching vibration appears along with the stretching vibrations of secondary amine and water of hydration peaks appeared around 3090 cm^−1^ to 3225 cm^−1^ region itself [24].

### 3.3. NMR spectral analysis

**Figure 2:**
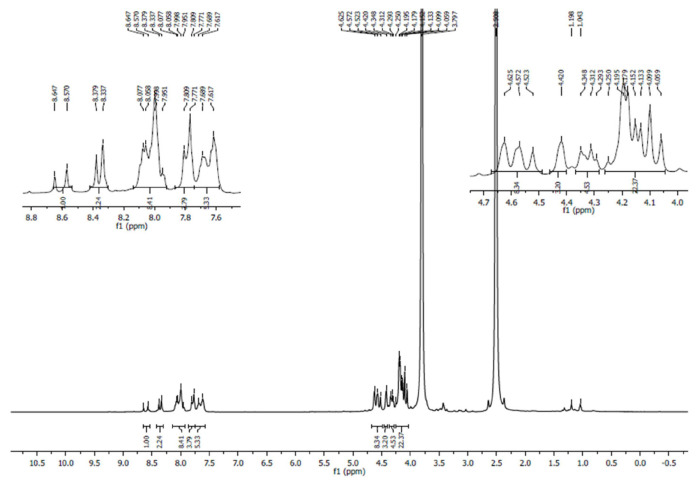
NMR spectrum of R1.

The peaks present in the ^1^H NMR spectrum of R1 (Figure 2) are assigned as mentioned here δ, (ppm); 8.58 (s, 2H, NCH), 7.62 (m, 4H, aromatic), 4.62 (m, 2H, cp subst), 4.29 (m, 2H, cp subst), 4.09 (s, 5H, cp unsubst), 2.05 (2s, 2H, CH_2_), 1.24 (s, 2H, 2NH). For R2 δ, (ppm); 8.03 (s, 2H, NCH), 7.81 (m, 4H, aromatic), 4.63 (m, 2H, cp subst), 4.25 (m, 2H, cp subst), 4.09 (s, 5H, cp unsubst), 2.50 (2s, 2H, CH_2_), 1.2 (s, 2H, 2NH) (Figure S4). The peaks present in the ^13^C NMR spectrum of R1 (Fig. S5) are assigned as mentioned here. δ, (ppm): 169.5 (C=O), 133.4 (C^4^, meta to CHO), 130.43 (C^5^, -CHO attached C), 128.2 (C^6^, -ortho to CHO), 127.9 (C^7^, -ortho to NO_2_, 2-nitrobenzaldehyde), 68.9 (C^3^, -α-carbon), 79.2 (C^2^, -CHO attached C), 70.1, (C^4^, -β-C ferrocenecarboxaldehyde), 39.28 (CH_2_, malonyldihydrazide, merged with DMSO peak).

### 3.4. Exploration of the captivity of metal ions

**Figure 3:**
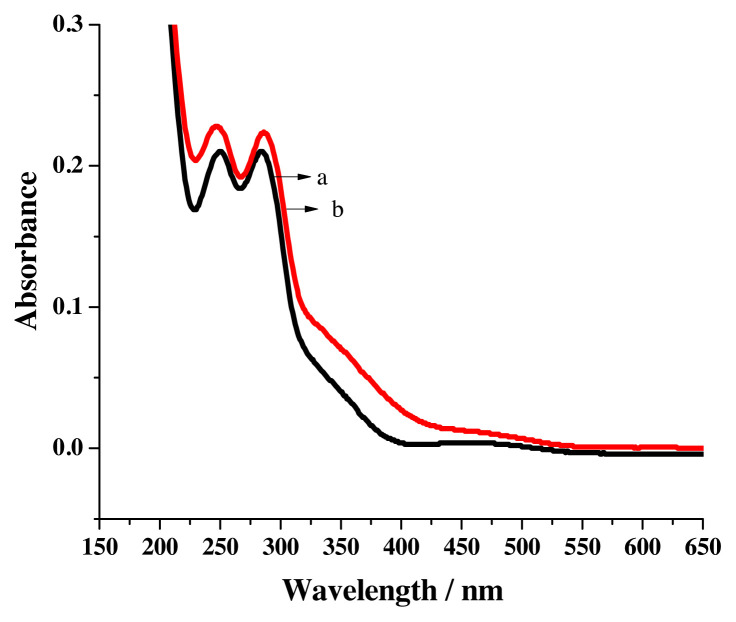
UV-visible spectrum of R1 in a) acetonitrile

The UV-visible spectrum of R1 in acetonitrile contains two peaks near 249 nm and 283 nm (Figure 3a) and in ethanol in addition to the two peaks seemed around 246 nm & 286 nm, a shoulder emerges close to 353 nm. (Figure 3b). Aromatic ring π-π* transition has been assigned [25] for the peaks and n-π* charge transfer transition has been allocated [26] for the shoulder that appeared in the near UV region.

**Figure 4:**
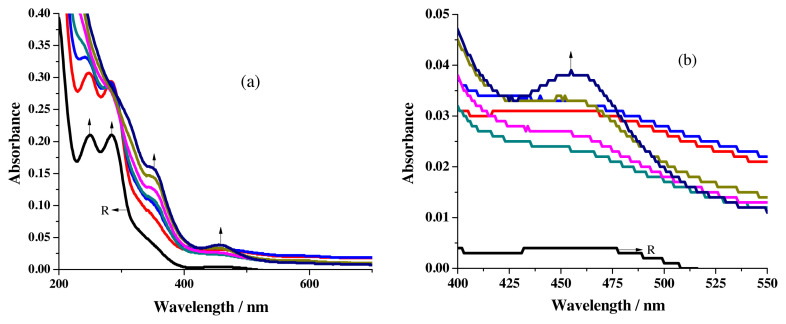
Spectral changes recorded for the addition of Cu^2+^ ions to R1 a) overall changes b) formation of MLCT band.

The difference in solubility of metal salts splits the sensing studies into two parts. Alcoholic solutions for MnCl_2_, Pb(OAc)_2_ &_,_ Cd(OAc)_2_ and acetonitrile solution for CuCl_2_, HgCl_2_ & NiCl_2_ were used. To conduct the titration studies in UV-visible spectrophotometer, 20 μL aliquot of 1.25 × 10^−3^ M metal salt solutions were added to 2.5 mL of 1 × 10^−5^ M receptor solution taken in the cuvette. Spectral changes noted for the addition of Cu^2+^ ions are given in Figure 4a. The development of a new peak around 457 nm (Figure 4b) is earmarked for the formation of a metal-to-ligand charge transfer band responsible for the combination of metal ions with receptor [25].

The disappearance of π-π* transition peaks with simultaneous formation of the shoulder around 348 nm also exposes the binding aptitude of R1 towards Cu^2+^ ions.

**Figure 5:**
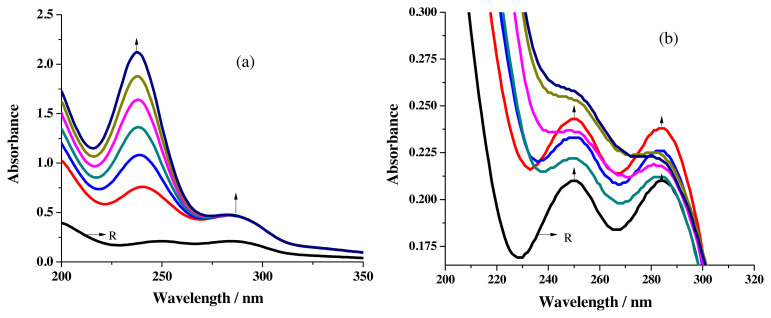
Changes perceived in the spectrum of R1 for the addition of a) Hg^2+^ ions b) Ni^2+^ ions, c) Pb^2+^ ions.

Generation of the new peak near 237 nm for the addition of Hg^2+^ ions (Figure 5a), the conversation of π-π* transition peaks into shoulders at the same wavelength upon increasing the concentration of Ni^2+^ ions (Figure 5b) and blue shift of ligand peaks (246 nm to 238 nm and 286 nm to 283 nm) for the hike of Pb^2+^ ion (Figure 5c) concentration expose the binding aptitude of R1. Cumulative addition of Cd^2+^ and Mn^2+^ ions increases absorbance value at the λ_max_ of sensor peaks for the first two additions and thereafter a decrease in absorbance was noticed.

**Figure 6:**
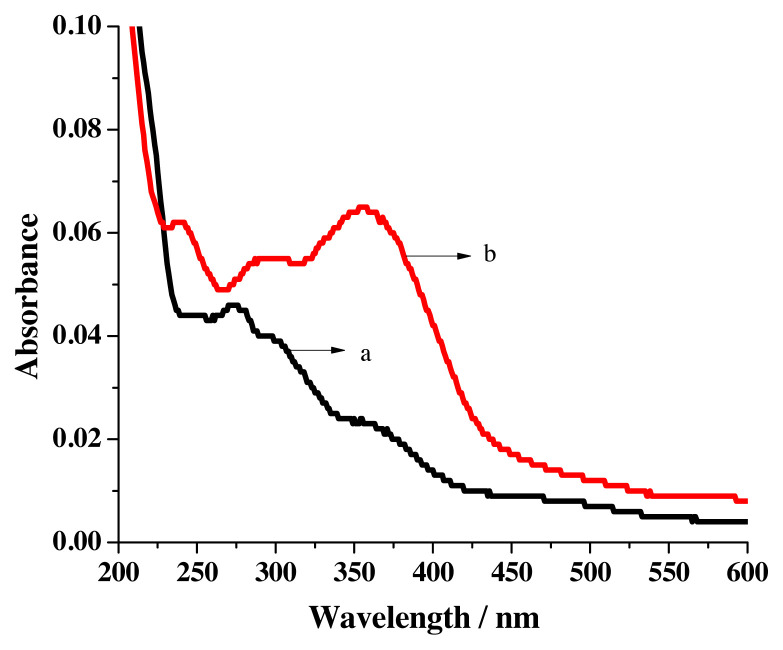
Electronic spectra of R2 in a) acetonitrile b) ethanol.

The electronic spectrum of R2 in acetonitrile (solvent) shows a peak at 272 nm and two shoulders around 300 nm & 363 nm (Figure 6a). In solvent ethanol two prominent peaks near 238 nm & 356 nm and a broad peak closer to 298 nm have been observed (Figure 6b). Observations made in the lower wavelength region have been assigned for π-π* transition and higher wavelength area have been allotted for n-π* transition [27].

**Figure 7:**
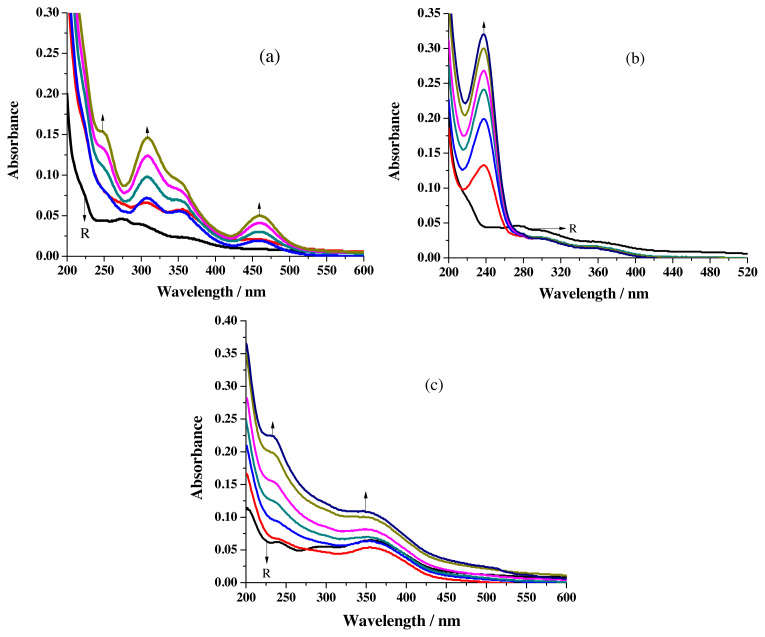
Formation of new peaks for the addition of a) Cu^2+^ b) Hg^2+^, c) Pb^2+^.

Coordination of R2 with Cu^2+^ ions is exposed by the generation of MLCT [25] absorption peak (459 nm) along with the development of new peak near 357 nm and a shoulder around 248 nm (Figure 7a). Incarceration of Hg^2+^ and Pb^2+^ ions by the receptor is revealed upon the formation of new peak at 237 nm (Figure 7b) and gradual disappearance of 298 nm broad peak (Figure 7c) respectively. Imprisonment of Ni^2+^ is exposed by the transformation of π-π* transition peaks into shoulder at the same wavelength for the addition of Ni^2+^ ions. Captivity of Cd^2+^ and Mn^2+^ is realized by the increase in absorbance value noticed in all wavelength regions for the successive addition of Cd^2+^ and Mn^2+^ ions.

### 3.5. Sensing analysis with electrochemical redox studies

**Figure 8:**
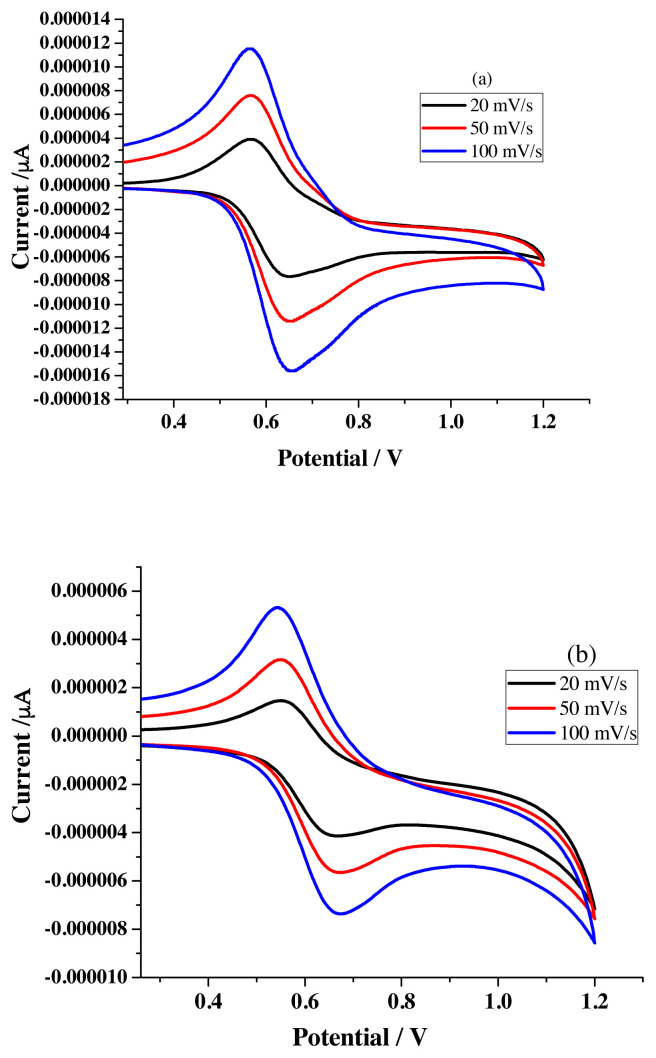
Voltammograms of R1 (1 × 10^−3^ M) in a) acetonitrile b) ethanol under different scan rate.

Sensing priority analysis is investigated against applied potential by comparing the electrochemical data obtained for metal free and metal added receptor solutions. Data extracted from the voltammograms (Figure 8a & 8b) recorded for R1 with different scan rate is associated with increased ΔE_P_, I_pa_ & I_pc_ values (Table 1). Noticed enhanced ΔE_p_ values (78–126 mV instead of 59 mV) emphasize the quasi-reversible one-electron redox behavior of ferrocene moiety [28].

**Figure 9:**
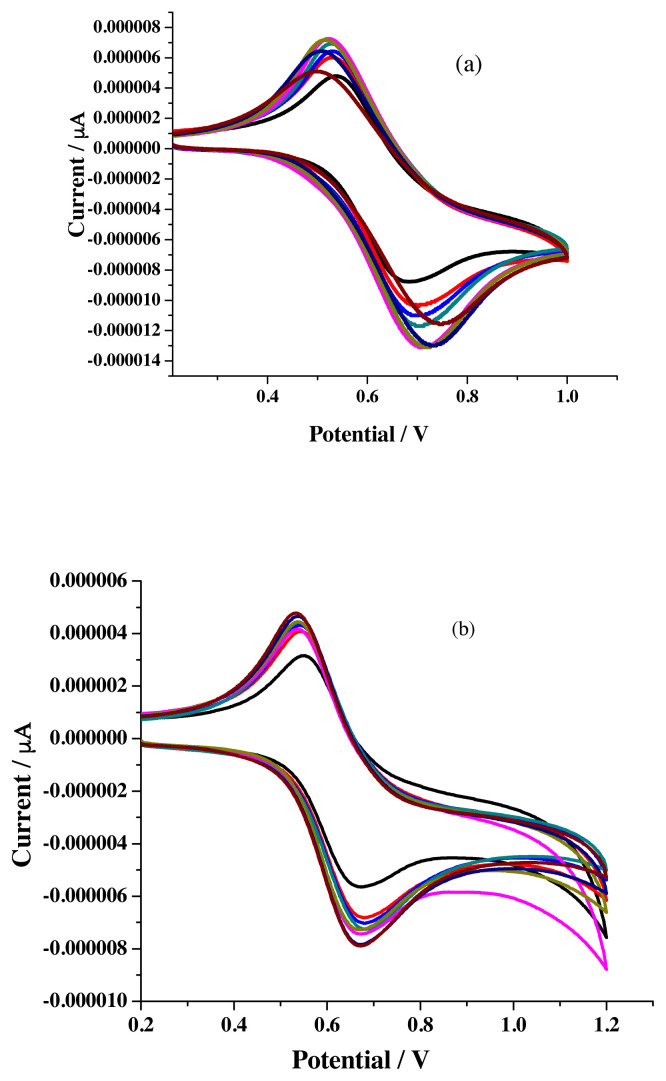
CV titration study of R1 (10^−3^ M) with different concentration of Pb^2+^ ions a) 10^−3^ M b) 10^−1^ M.

Voltammograms registered for the titration carried out in the three electrode cell compartment by keeping 10 mL of 1 × 10^−3^ M R1 solution and then increasing the concentration of metal salts solution by adding 20 μL aliquots of either 1 × 10^−3^ M (Figure 9a) or 1 × 10^−1^ M metal salt solutions (Figure 9b) possess positive potential shift for oxidation peak and negative potential shift for reduction peak which exposes the sensing ability of newly synthesized receptors towards various metal ion [29,30]. Figures 9a & 9b are the representative voltammograms (scan rate −50 mV/s) noticed for the addition of Pb^2+^ ions.

**Figure 10:**
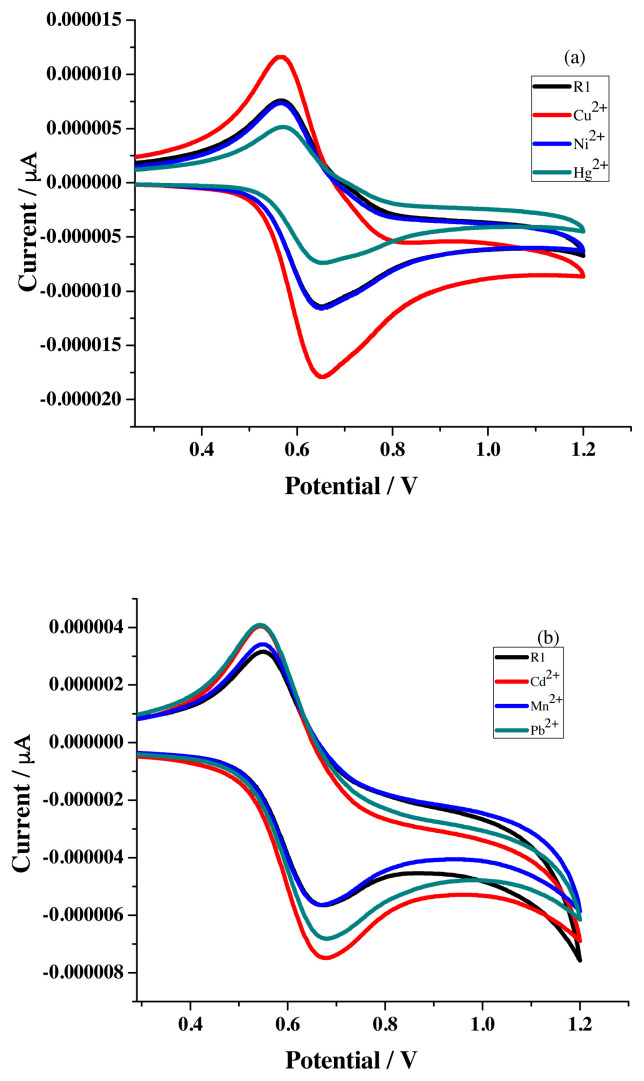
Voltammogram of R1 (1 × 10^−3^M) with different metal ions (1 x 10^−3^ M) in a) acetonitrile b) ethanol.

Cyclic voltammograms perceived for the addition of different metal ions solution (1 × 10^−3^ M) to the receptor solution with 1×10^−3^ M concentration are given in Figure 10a & 10b.

The difference in coordination ability of the various metal ions with the newly synthesized sensor compound is reflected in the observed varied amount of I_pa_ and ΔE_p_ values (Table 2) incurred from the electrochemical data. Further, the difference in the ΔI_pa_ ( %) calculated from the I_pa_ values noticed for the oxidation wave of receptor solution and different metal ion added receptor solution unearth the binding ability sequence of R1 as Cu-84.4 > Hg-30.3 > Cd-22.8 > Pb-22.8 > Mn-7.1 > Ni-2.83. It is proposed that the first factor for the difference in the obtained I_pa_ and ΔE_p_ values might be the variation in repulsive force operating between the oxidized ferrocene unit and sensed metal cations. The second factor might be the affinity acting between the sensor and metal ions [31].

**Figure 11:**
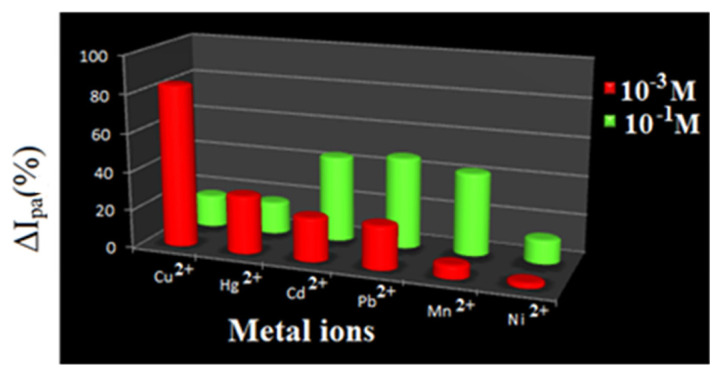
Comparison chart for sensing ability of R1.

In the CV titration with 1 × 10^−3^ M receptor solution and 1 × 10^−1^ M metal salts solution, the assessed percentage decrease in ΔI_pa_, which in turn estimated from anodic current (I_pa_) values noticed (Table 3) for the oxidation peak, reflects the sensing power of R1as Pb-47.4 > Cd-44.6 > Mn-42.7 > Cu-16.8 > Hg-16.8 > Ni-12.4. Dissimilar order of binding priority of R1 towards various metal ions under different concentrations unveil that R1 is more sensitive towards Cu, Hg & Cd at lower concentration and preferable binding at higher concentration is Pd, Cd & Mn (Figure 11).

**Figure 12:**
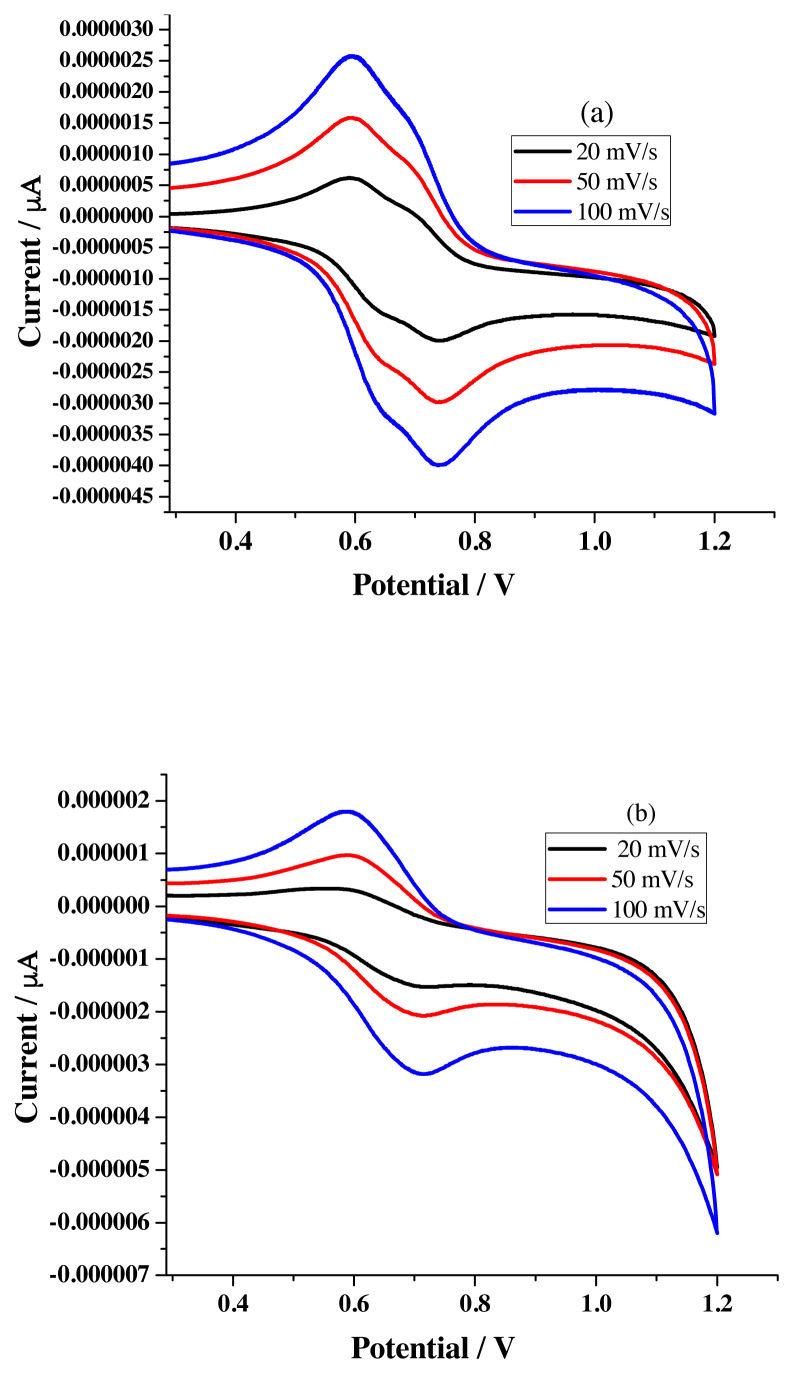
Voltammograms of R2 (1 × 10^−3^ M) in a) acetonitrile b) ethanol under different scan rate.

The I_pa,_ I_pc,_ and ΔE_P_ values noted for R2 (Table 4) with various scan rate (Figure 12a & 12b) follow the similar trend observed for R1 and reflect the quasi-reversible reduction process.

**Figure 13:**
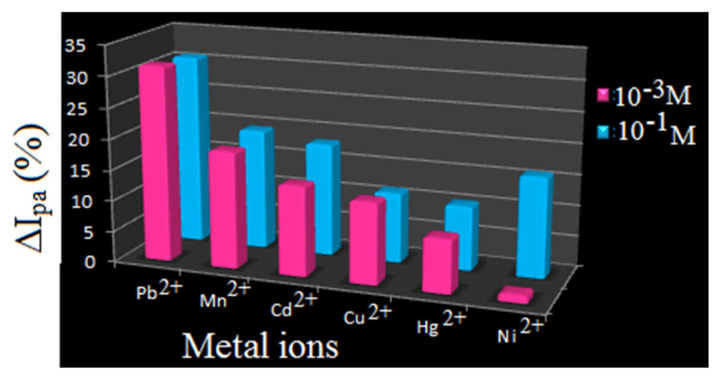
Comparison chart for sensing ability of R2.

Titration experiment for receptor R2 in CV studies was carried out by adopting the same procedure used for R1. Estimated percentage values of ΔI_pa_ expose that the order of binding under same molar additions is Pb > 31.6 > Mn-18.9 > Cd-14.5 > Cu-13.1 > Hg-8.6 > Ni-1.2 (Table 5) and multimolar accompaniment is Pb-30.9 > Mn-19.7 > Cd-18.3 > Ni-16.2 > Cu-11.4 > Hg-10.4 (Table 6). Appropriate metal ion concentration required by sensor R2 is pictured in Figure 13.

### 3.6. Antimicrobial studies

**Figure 14:**
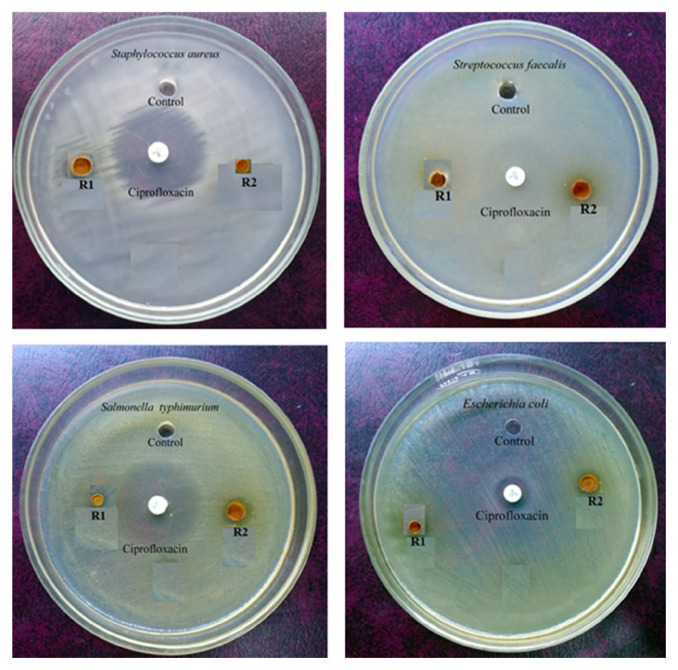
Activity against Staphylococcuse aureuse, Streptococcus faecalise, Salmonella typhimurium, and Escherichia coli.

**Figure 15:**
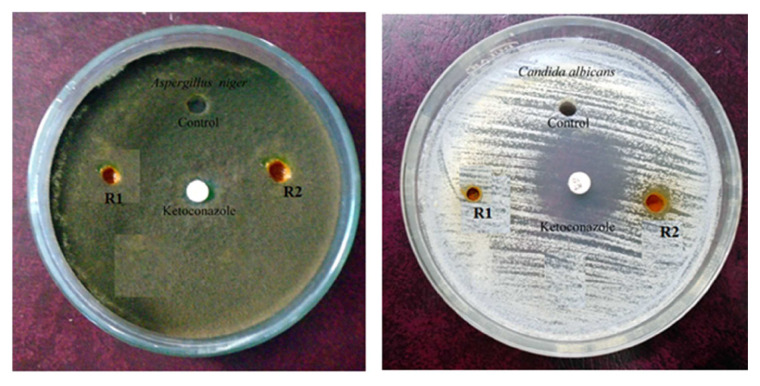
Antifungal activity against Aspergillus niger and Candida albicans.

The ability of the newly synthesized compounds to prevent the growth of bacteria was investigated against *Escherichia coli, Staphylococcus aureus*, *Salmonella typhimurium*, and *Streptococcus faecalise* in Mueller Hinton agar base by disc diffusion method (Figure 14). Likewise, the capability of R1 & R2 to restrict the progress of fungi was carried out in the base Sabouraud’s Dextrose agar for *Candida albicans* and *Aspergillus niger* (Figure 15). Average values of growth inhibition distances obtained from three different experiments are given in Table 7.

Compounds R1 and R2 avert the growth of fungus *Aspergillus niger* strongly to a level of 100% and 87.5% respectively like that of the standard material ketoconazole. Progression of *Candida albicans* is also prohibited up to 35%. Observed antibacterial activities are on par with ciprofloxacin (standard material).The above results demand more focused research by pharmacists to develop new formulations for fungi using the new compounds R1 and R2.

### 3.7. Molecular docking

**Figure 16:**
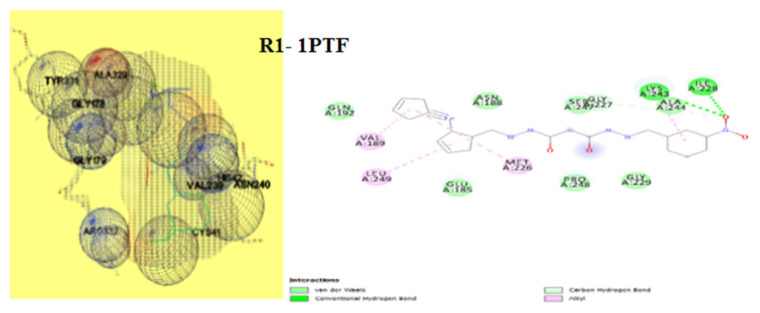

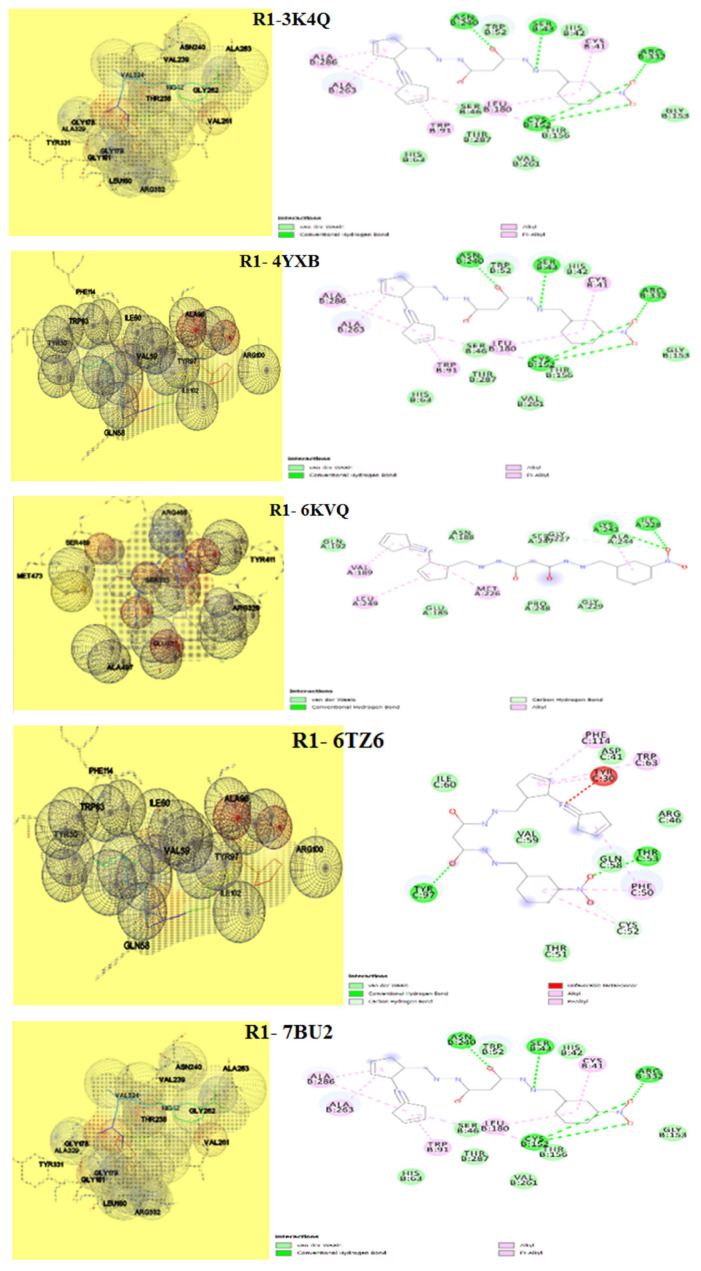
Docked 3D and 2D view of R1 with a) 1PTF, b) 3K4Q, c) 4YXB, d) 6KVQ, e) 6TZ6, f) 7BU2.

**Figure 17:**
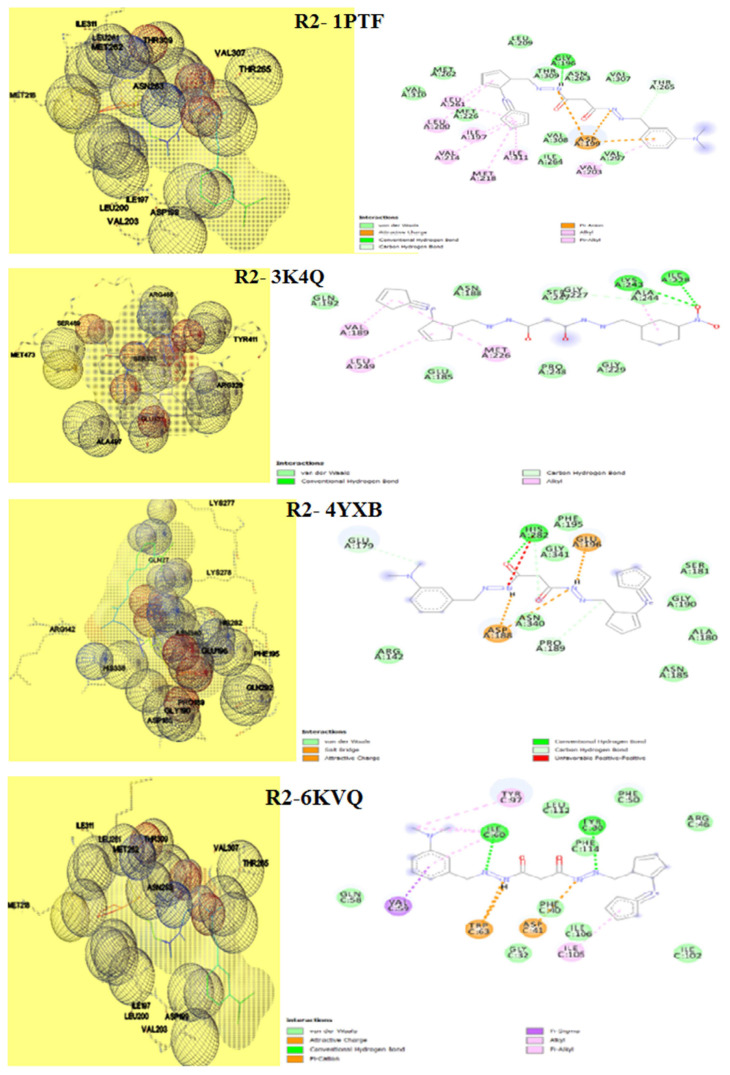

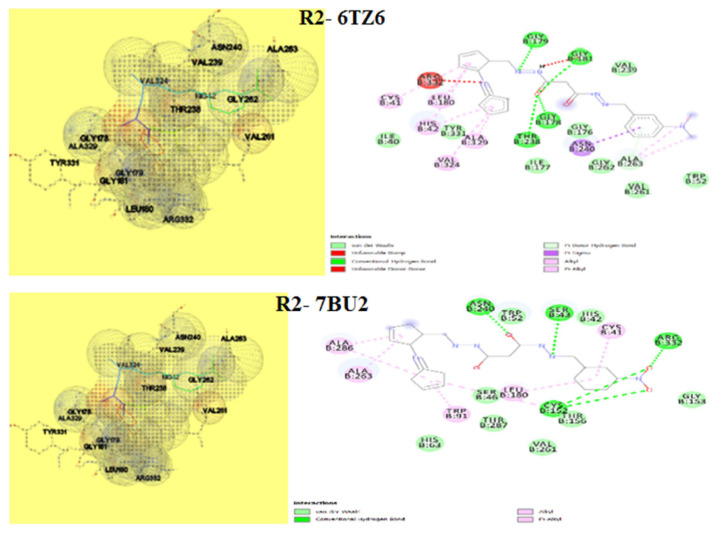
Docked 3D and 2D view of R2 with a) 1PTF, b) 3K4Q, c) 4YXB, d) 6KVQ, e) 6TZ6, f) 7BU2.

The way of interaction of the complex protein and ligand is investigated using molecular docking methods. Results obtained in the above analysis are given in Table 8. The 3D and 2D view binding of R1 & R2 are denoted in Figure 16 & 17, respectively. The binding scores of R1 fall between −4.21 to −8.62 Kcal mol^−1^ and R2 has the score values between −6.17 to −8.57 Kcal mol^−1^, which discloses that the sensor can interact positively with the protein of microorganisms. Sensor R1 showed the highest binding affinity with fungi proteins 3K4Q (−8.62 Kcal mol^−1^), 6TZ6 (−8.22 Kcal mol^−1^), and bacteria protein 7BU2 (−7.32 Kcal mol^−1^). Likewise sensor R2 displayed better binding score for fungi proteins 3K4Q (−7.62 Kcal mol^−1^), 6TZQ (−7.12 Kcal mol^−1^) and bacteria proteins 6KVQ (−8.57 Kcal mol^−1^) & 7BU2 (−7.67 Kcal mol^−1^). Both sensors (R1 & R2) make H-bonds with active site residue 282-HIS, 246-ILE, 97-TYR, 105-ILE, 332-ARG, 329-ALA, 209-LEU, and 91-TRP. Data obtained above exposes that both compounds R1 and R2 are of a gifted category for further formulation of a new class of antifungal agents and demands more investigation by pharmacists.

## 4. Conclusion

Our attempts to prepare Schiff bases having ferrocenecarboxaldehyde attached with imine on one side and aromatic aldehyde connected azomethine group on the other side have resulted with N’^1^-((E)-ferrocenylidene)-N’^3^-((E)-2-nitrobenzylidene) malonohydrazide and N’^1^-((E)-ferrocenylidene)-N’^3^-((E)-2-hydroxy-5-nitrobenzylidene))malonohydrazide. Spectral investigations using FTIR, ^1^HNMR, and Mass validate the development of above said unsymmetrical compounds. Titration studies coupled with electronic spectral analysis disclose that the newly synthesized sensors are capable of sensing different metal ions. Voltammograms recorded for different metal ions added receptor solution also reveal the competency of receptors to recognize various metal ions. The ΔI_pa_ (%) values estimated from the I_pa_ values noticed for the metal ions added receptor solution divulge the modest amount of metal ion concentration required for effective sensing. The enhanced antifungal activity identified for the newly synthesized compounds R1 & R2 in, in vitro analysis and noticed high H-bond formation binding energy towards proteins of fungi in molecular docking studies goad further research by the pharmacist to develop a new formulation of antifungal agents incorporating the new compounds reported in this article.

S1 Mass spectrum (m/z) of R1.

S2 Mass spectrum (m/z) of R2.

S3 FTIR spectrum of R2.

S4 ^1^H NMR spectrum of R2.

S5 ^13^C NMR spectrum of R2.

## Figures and Tables

**Table 1:** Cyclic voltammetry data of R1 (1 × 10^−3^ M) with different scan rates.

Scan Rate- mV/s	E_pa_ (V)	E_pc_ (V)	ΔE_p_ (V)	E_1/2_ (V)	I_pa_ × 10^−5^ (μA)	I_pc_ × 10^−6^ (μA)
Solvent–Acetonitrile						
20	0.646	0.566	0.08	0.606	−0.774	4.005
50	0.646	0.568	0.078	0.607	−1.149	7.515
100	0.651	0.566	0.085	0.608	−1.572	11.588
Solvent–Ethanol						
20	0.663	0.548	0.115	0.605	−4.132	1.516
50	0.674	0.548	0.126	0.611	−5.702	3.176
100	0.669	0.546	0.123	0.607	−7.437	5.379

**Table 2:** Cyclic voltammetry data of R1 (1 × 10^−3^ M) with different metal ions (1 × 10^−3^M) under 50 mV/s scan rate.

Addition	E_pa_ (V)	E_pc_ (V)	ΔE_p_ (V)	E_1/2_ (V)	I_pa_ × 10^−5^ (μA)	I_pc_ × 10^−6^ (μA)
Solvent–Acetonitrile						
Receptor	0.646	0.568	0.078	0.607	−1.149	7.515
Cu^2+^	0.646	0.565	0.081	0.605	−1.806	1.170
Ni^2+^	0.644	0.565	0.079	0.604	−1.161	7.302
Hg^2+^	0.648	0.572	0.076	0.61	−7.489	5.234
Solvent–Ethanol						
Receptor	0.674	0.548	0.126	0.611	−5.702	3.176
Cd^2+^	0.673	0.541	0.132	0.607	−7.521	4.117
Mn^2+^	0.669	0.545	0.124	0.607	−5.670	3.419
Pb^2+^	0.675	0.541	0.134	0.608	−6.856	4.117

**Table 3:** Cyclic voltammetry data for R1 (1 × 10^−3^ M) with different metal ions (1 × 10^−1^M) under 50 mV/s scan rate.

Addition	E_pa_ (V)	E_pc_ (V)	ΔE_p_ (V)	E_1/2_ (V)	I_pa_ × 10^−5^ (μA)	I_pc_ × 10^−6^ (μA)
Solvent–Acetonitrile						
Receptor	0.646	0.568	0.078	0.607	−1.149	7.515
Cu^2+^	0.661	0.565	0.096	0.613	−1.335	9.033
Ni^2+^	0.649	0.561	0.088	0.605	−1.403	8.585
Hg^2+^	0.663	0.555	0.108	0.609	−1.388	9.033
Solvent–Ethanol						
Receptor	0.674	0.548	0.126	0.611	−5.702	3.176
Cd^2+^	0.672	0.536	0.136	0.604	−8.439	5.741
Mn^2+^	0.693	0.534	0.159	0.613	−9.589	5.542
Pb^2+^	0.698	0.529	0.169	0.616	−1.033	6.041

**Table 4:** Cyclic voltammetry data of R2 (1 × 10^−3^ M) with different scan rates.

Scan Rate- mV/ sec	E_pa_ (V)	E_pc_ (V)	ΔE_p_ (V)	E_1/2_ (V)	I_pa_ × 10^−6^ (μA)	I_pc_ × 10^−6^ (μA)
Solvent –Acetonitrile						
20	0.739	0.586	0.153	0.662	−1.988	0.648
50	0.739	0.593	0.146	0.666	−3.046	1.607
100	0.739	0.595	0.144	0.667	−4.025	2.604
Solvent–Ethanol						
20	0.698	0.605	0.093	0.651	−1.534	0.290
50	0.712	0.594	0.118	0.653	−2.105	0.953
100	0.714	0.588	0.126	0.651	−3.219	1.809

**Table 5:** Cyclic voltammetry data of R2 (1 × 10^−3^ M) with different metal ions (1 × 10^−3^M) under 50 mV/ s scan rate.

Addition	E_pa_ (V)	E_pc_ (V)	ΔE_p_ (V)	E_1/2_ (V)	I_pa_ × 10^−6^ (μA)	I_pc_ × 10^−6^ (μA)
Solvent–Acetonitrile						
Receptor	0.739	0.593	0.146	0.666	−3.046	1.607
Cu^2+^	0.739	0.589	0.15	0.664	−3.440	1.396
Ni^2+^	0.741	0.595	0.146	0.668	−2.991	1.605
Hg^2+^	0.727	0.572	0.155	0.649	−2.856	1.621
Solvent–Ethanol						
Receptor	0.712	0.594	0.118	0.653	−2.105	0.953
Cd^2+^	0.708	0.578	0.13	0.643	−2.258	1.115
Mn^2+^	0.715	0.580	0.135	0.647	−2.619	1.176
Pb^2+^	0.706	0.574	0.132	0.64	−2.419	1.394

**Table 6:** Cyclic voltammetry data for R2 (1 × 10^−3^ M) with different metal ions (1× 10^−1^ M) under 50 mV/s scan rate.

Addition	E_pa_ (V)	E_pc_ (V)	ΔE_p_ (V)	E_1/2_ (V)	I_pa_ × 10^−6^ (μA)	I_pc_ × 10^−6^ (μA)
Solvent–Acetonitrile						
Receptor	0.739	0.593	0.146	0.666	−3.046	1.607
Cu^2+^	0.702	0.555	0.147	0.625	−3.707	1.814
Ni^2+^	0.715	0.562	0.153	0.638	−3.481	1.919
Hg^2+^	0.708	0.559	0.149	0.633	−2.677	1.795
Solvent–Ethanol						
Receptor	0.712	0.594	0.118	0.653	−2.105	0.953
Cd^2+^	0.721	0.578	0.143	0.649	−2.865	1.167
Mn^2+^	0.706	0.559	0.147	0.632	−2.165	1.187
Pb^2+^	0.719	0.576	0.143	0.647	−2.461	1.379

**Table 7:** Data obtained in in vitro antimicrobial analysis.

S. no	Microorganisms	Control	R1	R2	Ciprofloxacin/ Ketoconazole
zone of inhibition in mm for bacteria					
1	*Staphylococcus aureus*	-	07	07	25
2	*Streptococcus faecalis*	-	-	05	24
3	*Escherichia coli*	-	06	06	12
4	*salmonella typhimurium*	-	09	09	27
**zone of inhibition in mm for fungi**					
1	*Candida albicans*	-	09	09	25
2	*Aspergillus niger*	-	08	07	08

**Table 8:** Results gained from molecular docking studies.

	Free binding energy, kcal mol^−1^		R1		R2	
PDB	R1	R2	Hydrogen bonds with receptor amino acids	Distance(Å)	Hydrogen bonds with receptor amino acids	Distance(Å)

1PTF	−4.21	−6.72	3-LYS	3.40	194-TYR	3.32
			7-HIS	3.45	195-LEU	3.43
				1.84	214-ASP	3.52
			87-ALA	4.85	216-ILE	3.43

3K4Q	−8.62	−7.62	179-GLU	3.97	216-ALA	3.78
					219-PRO	3.72
			282-HIS	3.24	246-ILE	3.43
			340-ASN	3.95	253-ILE	3.92

4YXB	−6.72	−6.17	26-ALA	3.18	26-ALA	3.47
			29-PRO	3.89	29-PRO	3.43
			46-ILE	3.97	46-ILE	3.58
			53-ILE	3.64	53-ILE	3.43

6KVQ	−7.02	−8.57	197-ILE	3.70	52-TRP	3.78
			199-ASP	3.76	91-TRP	3.27
			200-LEU	3.69	156-THR	3.43
			203-VAL	3.41	180-LEU	3.92
			209-LEU	3.12		

6TZ6	−8.22	−7.12	40-PHE	3.93	60-ILE	3.80
			41-ASP	3.69	97-TYR	3.94
			59-VAL	3.88	105-ILE	3.38
			60-ILE	3.98	114-PHE	3.50
			97-TYR	3.23		

7BU2	−7.32	−7.67	180-LEU	3.67		
			236-VAL	3.49	324-VAL	4.00
			240-ASN	3.98	329-ALA	3.36
			324-VAL	3.74	332-ARG	3.58
			329-ALA	3.30		
			332-ARG	3.09		
