# Supplementary figures and images for: Synthesis of polydentate, multi metal ion sensing, unsymmetrical Schiff bases with complimented antifungal activity
Source: Turk J Chem. 2022 Mar 1;46(4):1024–41. doi: 10.55730/1300-0527.3412 (PMC10395746; doi:10.55730/1300-0527.3412)

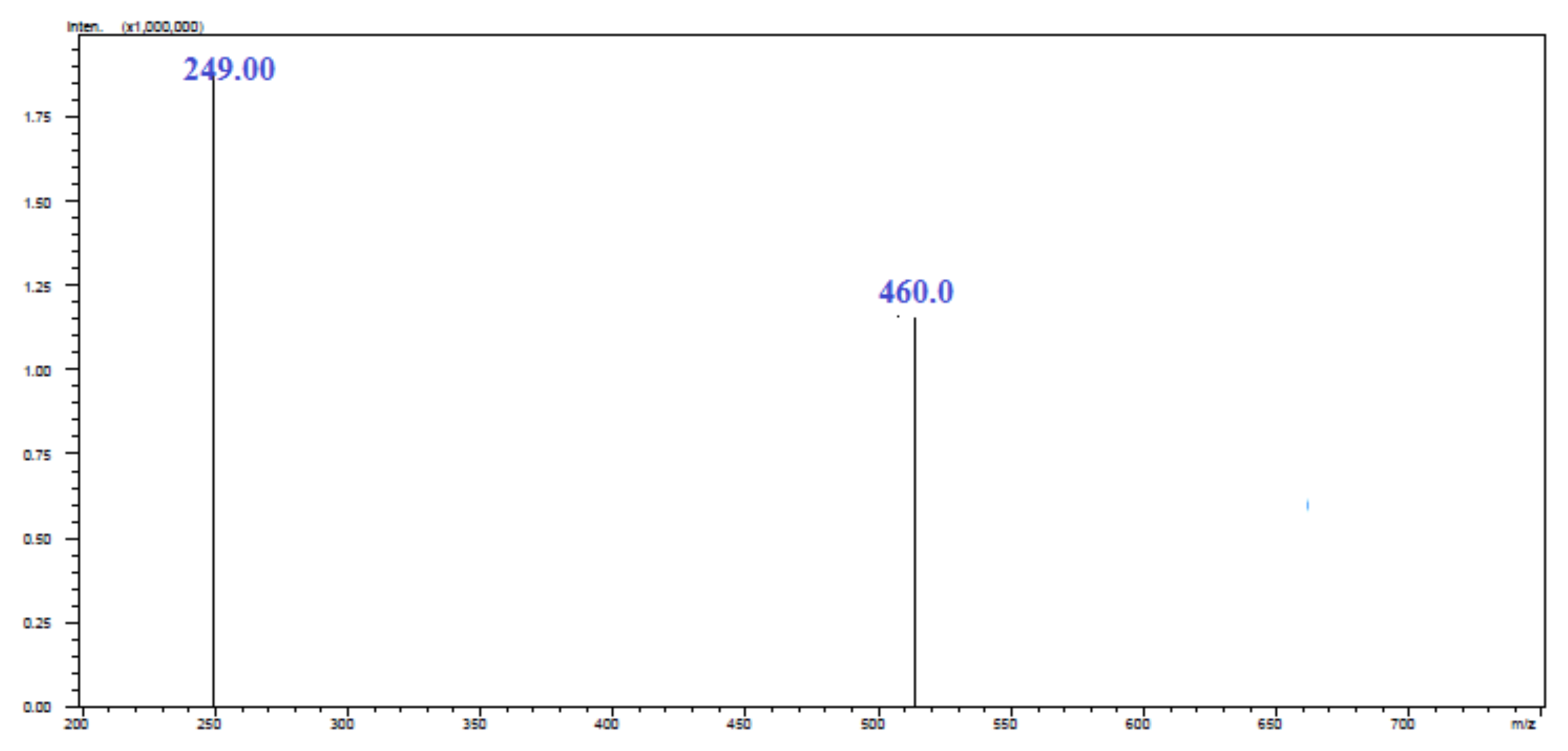

Supplement: S1 — Mass spectrum (m/z) of R1. [file turkjchem-46-4-1024s1.tif]

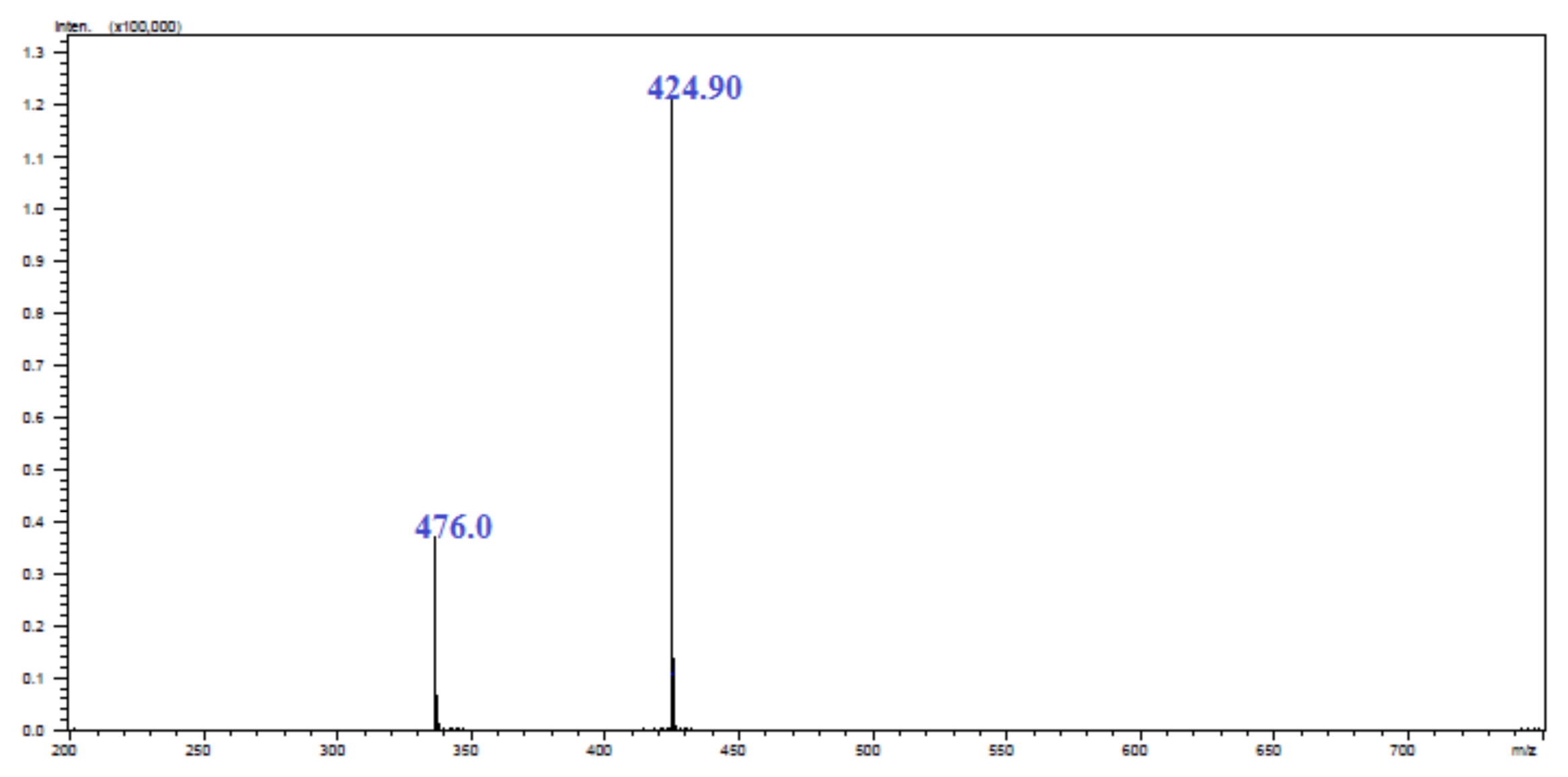

Supplement: S2 — Mass spectrum (m/z) of R2. [file turkjchem-46-4-1024s2.tif]

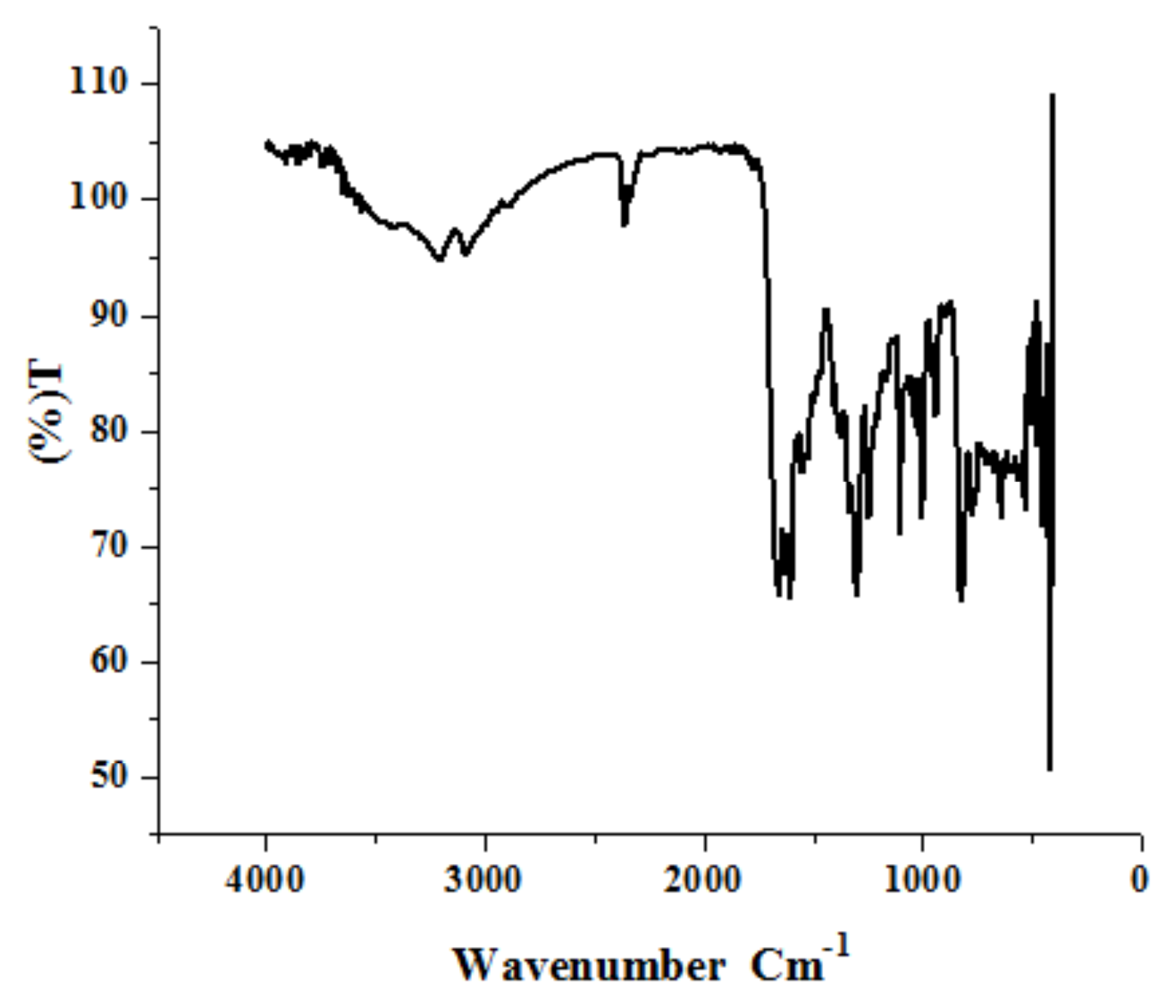

Supplement: S3 — FTIR spectrum of R2. [file turkjchem-46-4-1024s3.tif]

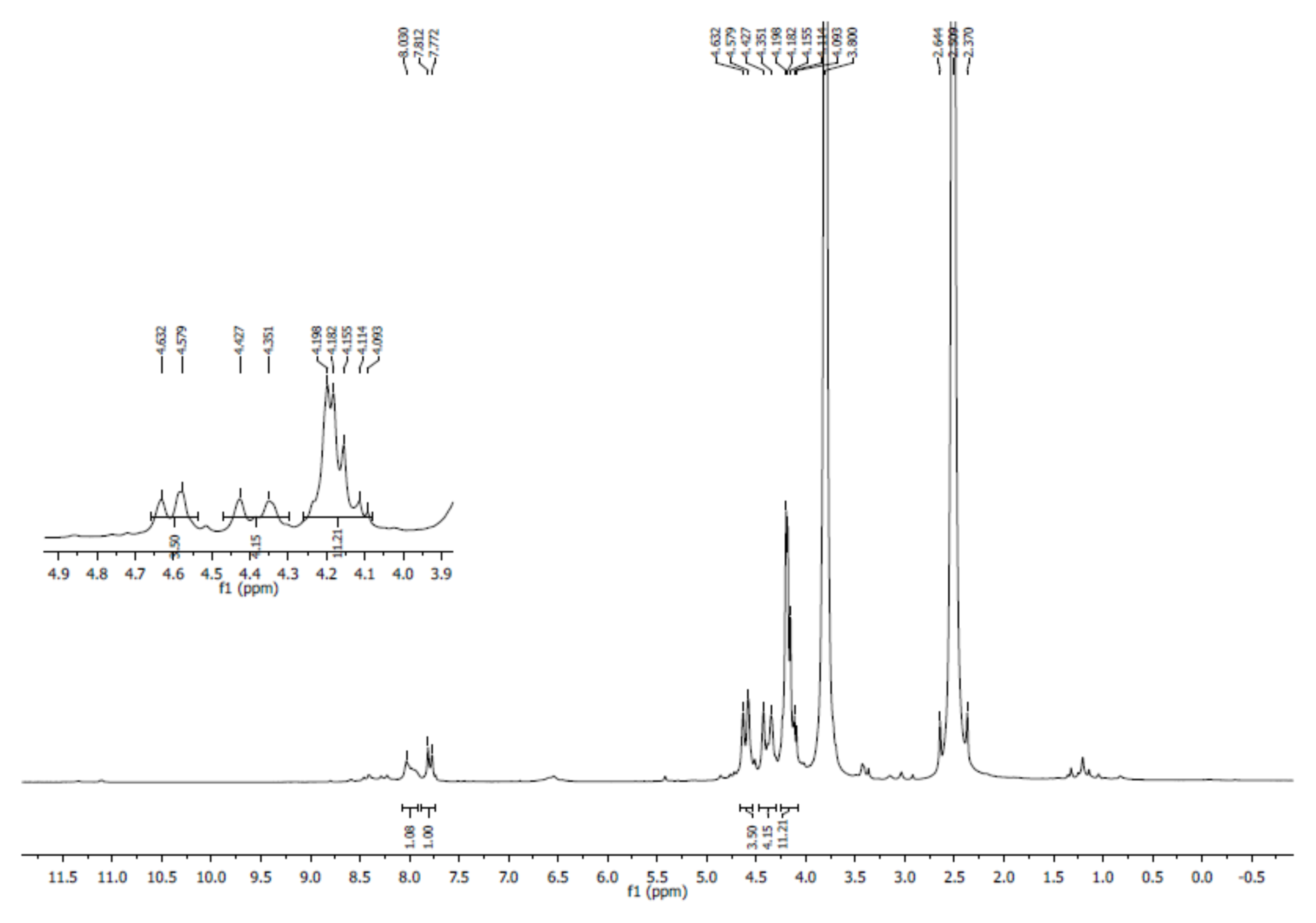

Supplement: S4 — 1H NMR spectrum of R2. [file turkjchem-46-4-1024s4.tif]

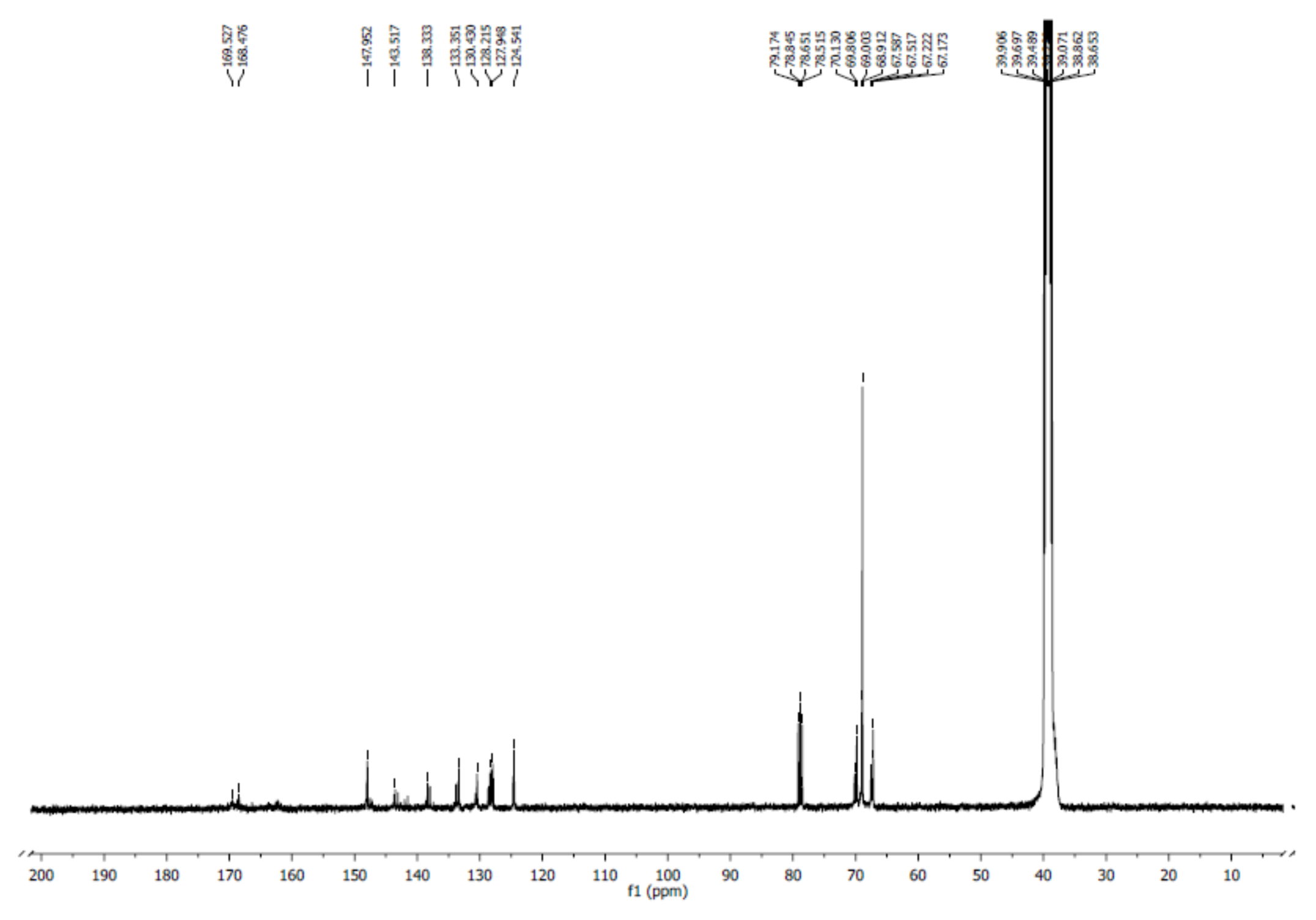

Supplement: S5 — 13C NMR spectrum of R2. [file turkjchem-46-4-1024s5.tif]
